# Q fever immunology: the quest for a safe and effective vaccine

**DOI:** 10.1038/s41541-023-00727-6

**Published:** 2023-09-07

**Authors:** Gayathri Sam, John Stenos, Stephen R. Graves, Bernd H. A. Rehm

**Affiliations:** 1 Centre for Cell Factories and Biopolymers, Griffith Institute for Drug Discovery, Griffith University, Brisbane, QLD 4111 Australia; 2 Australian Rickettsial Reference Laboratory, University Hospital, Geelong, VIC 3220 Australia; 3 Elizabeth Macarthur Agricultural Institute, NSW Department of Primary Industries, Menangle, NSW 2567 Australia; 4 Menzies Health Institute Queensland, Griffith University, Gold Coast, QLD 4222 Australia

**Keywords:** Bacterial infection, Vaccines

## Abstract

Q fever is an infectious zoonotic disease, caused by the Gram-negative bacterium *Coxiella burnetii*. Transmission occurs from livestock to humans through inhalation of a survival form of the bacterium, the Small Cell Variant, often via handling of animal parturition products. Q fever manifests as an acute self-limiting febrile illness or as a chronic disease with complications such as vasculitis and endocarditis. The current preventative human Q fever vaccine Q-VAX poses limitations on its worldwide implementation due to reactogenic responses in pre-sensitized individuals. Many strategies have been undertaken to develop a universal Q fever vaccine but with little success to date. The mechanisms of the underlying reactogenic responses remain only partially understood and are important factors in the development of a safe Q fever vaccine. This review provides an overview of previous and current experimental vaccines developed for use against Q fever and proposes approaches to develop a vaccine that establishes immunological memory while eliminating harmful reactogenic responses.

## Brief history and background

The emergence of an unknown abattoir fever in Brisbane (Australia) preceded the discovery of *Coxiella burnetii* as the causative agent of this illness. In 1938 Derrick was assigned the task of unraveling the mystery behind an unknown febrile illness among abattoir workers in Brisbane. Despite its clinical resemblance to other known diseases, Derrick was convinced it was a new type of fever and denoted it “Q (for query) fever”. Despite his success in transmitting the disease to guinea pigs from blood and urine of infected patients, he was unable to isolate the pathogen and speculated that the causative agent was a virus^1^. The infected tissue containing the “Q fever virus” was sent to Burnet who determined its structure to be similar to a *Rickettsia* species. Concurrently Cox and Davis isolated an infectious agent from ticks in Montana (USA). They demonstrated that this agent was a minute Gram-negative pleomorphic *Rickettsia*-like organism^2^ and later demonstrated that it could grow in chicken embryo cultures^3^. The realization that these two discoveries involved the same organism dawned, when Dyer contracted the illness a few days after his stay at the Montana lab with Cox. Later, the organism was renamed “*Coxiella burnetii*” in honor of Cox and Burnets’ seminal work in identifying this infectious agent.

*C. burnetii*, the etiological agent of Q fever, causes a flu-like infectious zoonotic disease of worldwide importance^4,5^. *C. burnetii* reservoirs in ruminant livestock such as goats, sheep, and cattle who transmit the bacterium via their parturition products. Transmission occurs through direct contact with infected animals or their products of parturition via inhalation of the *C. burnetii* small cell variant (SCV). For this reason, people who reside in livestock areas and handle livestock are susceptible to Q fever, making it a disease of location and occupation^5–7^. Owing to its excellent environmental stability and low infectious dose, *C. burnetii* is considered to be a category B agent of bioterrorism by the Centre for Disease Control and Prevention^8^. The disease has been a substantial cause of morbidity in Australia, with New South Wales and Queensland reporting most cases^9–11^. Q fever is also reported worldwide, and the most notable epidemic was the major outbreak in the Netherlands from 2007–2010 involving 4026 cases^12^.

### *Coxiella burnetii*

*C. burnetii* is a pleomorphic Gram-negative bacterium that belongs to the gamma-subgroup of the *Proteobacteriacae*, order *Legionellales*, family *Coxiellaceae*, genus *Coxiella* and species *C. burnetii*^13^*. C. burnetii* exhibits morphological and antigenic variation. The morphological forms arise from its biphasic developmental cycle and consist of the SCV and the large cell variant (LCV). The SCV is a short rod-shaped, non-replicative extracellular form of *C. burnetii*. Its structure enables survival under harsh environmental conditions and assists dissemination^9,14^. As the SCV, *C. burnetii* invades host cells and gradually develops into the LCV. LCVs are pleomorphic and the metabolically active forms that are capable of replication inside eukaryotic cells^14,15^.

The phase variations of *C. burnetii* include virulent phase I and avirulent phase II cells^16^. The difference between the two-phase variants lies in the structure of the lipopolysaccharide (LPS), a major component of the bacterial cell wall. The full-length LPS (smooth) of the phase I variant consists of Lipid A, inner and outer core with an O-antigen that contains two unique sugars, virenose and dihydrohydroxystreptose^17^. Upon serial passages in embryonated eggs, cell cultures, and synthetic media, phase I cells gradually transition to avirulent phase II, which consists of a truncated (rough) LPS that only retains the inner core of oligosaccharides and lipid A^18–20^. As *C. burnetii* is highly infectious, causes consistent disabilities, survives harsh environmental conditions, and can be mass-produced for aerosol transmission, it has been classified as a category B bioterrorist agent. This led to operation Whitecoat carried out by the U.S Army where defensive measures to Q fever, including vaccine candidates, were tested by exposing war-time conscientious objectors to the pathogen^21^. An aerosolization chamber was used to test volunteers vulnerability to aerosolized pathogens^22^. While some of the volunteers became sick, they all fully recovered and no death were reported. In the 1990s, a Japanese religious group that used sarin gas to attack Tokyo was known to develop *C. burnetii* for intentional release^23^.

## Pathogenesis of Q fever

The pathogenesis of Q fever is a convergence of *C. burnetii* virulence with host cell resistance^24^. Following inhalation of SCVs, *C. burnetii* primarily targets alveolar macrophages. They are internalized by both phagocytotic and non-phagocytotic cells^25,26^. Phase I and phase II enter phagocytic cells through the engagement of different receptors. The uptake of *C. burnetii* by non-phagocytotic cells is through a “zippering” mechanism. The interaction of surface ligands with cognate receptors on host cells such as fibroblasts^8^ and HeLa cells^27^ passively encloses them in a zipper-like manner, accompanied by remodeling of the actin cytoskeleton^25,27,28^. Recent studies by multi-phenotypic high-content screening identify OmpA as an essential PAMP of *C. burnetii* that enables its entry into non-phagocytic cells^28^. Complement receptor CR3 and leukocyte response integrin (α_v_β_3_) are receptors present on monocytes, involved in the uptake^29^. The avirulent variant is readily recognized by both CR3 and integrin receptors after which it is efficiently eliminated following phagocytosis. In contrast phase I uptake is mediated by α_v_β_3_ alone^29–31^_._ This difference in receptor recognition lies in the LPS structure variation. The resistance of the phase I variant to serum complement-mediated attack^32^ suggests that steric hindrance provided by LPS might mask its opsonization by the complement system, hence the lack of recognition by its cognate CR3 receptor. Furthermore, impaired spatial rearrangement of CR3 outside membrane protrusions inhibits its essential crosstalk with the integrin receptor resulting in impaired uptake of phase I bacteria^30^. Internalization of phase I C. *burnetii* by the integrin receptor α_v_β_3,_ initiates extensive membrane ruffling through rearrangement of the filamentous (F)-actin cytoskeleton controlled by the Rho family of GTPases^33,34^. Once contained in the early phagosome, now referred to as a “*Coxiella* containing Vacuole” (CCV), it fuses with early endosomes. This process is similar to the usual canonical pathway and regulated by GTPase Rab5, which recruits early endosome antigen EEA1 that promotes the fusion of the endosome with the CCV^35^. The maturing CCV increases in size as it enters the late phagosome stage^25,26^. Rab7 replaces Rab5, while vacuolar ATPase on the CCV membrane pumps protons into the CCV reducing the pH to 5.5–6. Lysosome associated membrane glycoprotein (LAMP) LAMP1 and LAMP 2 are recruited to the late endosome stage^35,36^. The phagosomes that house phase II C. *burnetii* transition to a phagolysosome where it is destroyed by the action of lysosomal enzymes such as cathepsins and hydrolases^7^. Remarkably, studies with THP-1 cells demonstrate that vacuoles that shelter virulent *C. burnetii* do not acquire lysosomal markers and therefore do not transition to a phagolysosome, escaping their degradation^37^. Contradicting this theory, later studies reveal that fusion of CCV containing the virulent phase I with lysosome does occur but is halted due to its interaction with the autophagy pathway. The overexpression of the autophagic proteins GFP-LC3 or GFP-Rab24 increases CCV maturation after early infection^38–40^. The CCV membrane equips itself with LC3 markers a few minutes after infection^41^. The fusion of lysosome protein with CCV is further delayed by starvation-induced autophagy^40^. Autophagy is a homeostatic process that degrades and recycles molecular components of damaged organelles and proteins inside a cell. It involves the recruitment of Atg8 proteins such as LC3, which enclose the ubiquitinated cargo in the autophagosome. Fusion with the lysosome to generate the autophagolysosome mediates the degradation of the cargo by lysosomal enzymes^42,43^. The CCV is known to interfere with the host autophagic pathway^44^ to favor its enlargement and survival inside the host cell^40^. Diverted phagolysosomal maturation is speculated to provide time for the differentiation of metabolically inactive SCV inside the CCV to the metabolically active LCV form^39,45^ and the autophagy cargo acts as a source of nutrients to initiate replication and metabolic development to LCV^26^. The transition of SCV to LCV in mature CCVs halts the late CCV endosome maturation, retains the LC3 markers, and acquires the lysosomal glycoproteins LAMP1, and LAMP2^26^. The acidic pH in the CCV lumen sustains their survival as acidity is required for the assimilation of nutrients required for the synthesis of nucleic acids and amino acids^46^. Homotypic fusions with multiple small CCVs and heterotypic fusions with autophagic vesicles result in a large parasitophorous vacuole (PV) that occupies half the volume of the cell. The PV is surrounded by a cholesterol-rich membrane and lipid raft proteins flotillin-1 and flotillin-2 that enables membrane fusions^39^ and is filled with LCV and SCV variants. Six days post-infection the LCV reverts back to the SCV form, retaining the features of late CCV^25^. The fate of the host cell at this point is exploited in two ways by *C. burnetii:* (1) it can inhibit apoptosis by actively inhibiting signaling pathways^47^ or (2) Induction of pro-survival factors such as the ERKI, ERK2, and AKT family^48^. The need for prolonged survival of host cells could be crucial for the establishment of chronic disease and continued survival of *C. burnetii*. Similarly, *C. burnetii* can also initiate apoptosis of invaded host cells in a caspase-independent pathway, disseminating replicating bacteria to infect other susceptible cells^49^.

## Host immune responses

One characteristic of the facultative intracellular *C. burnetii* is its ability to survive and replicate in the acidic niche of phagocytes^50^. This makes them inaccessible to circulating antibodies and the complement system. Cell-mediated responses are required for the elimination and control of infection. While the acute infection is sometimes controlled by innate mechanisms, host responses to chronic infection require a more specific adaptive arm of the immune system^51,52^.

### Innate responses to *C. burnetii*

Innate responses are mediated by monocytes, macrophages and natural killer (NK) cells. Monocytes and macrophages, which are the primary targets, have differential responses depending on the variant that invades the cell. Invasion by phase II can be readily controlled by innate phagocytic function, whereas phase I *C. burnetii* requires the combined forces of innate and cell-mediated responses to eliminate them^50,53^.

### Monocytes and macrophages

The interaction of *C. burnetii* with macrophages and monocytes induces their polarization into the M1 or M2 macrophage phenotype. During acute infection, circulating monocytes polarize toward the M1 phenotype, which secretes the cytokines IFN-γ, IL-6, IL-12, and expresses CCR7^54^. The upregulated expression of nitric oxide synthase (NOS) catalyzes the conversion of L-arginine to citrulline to produce reactive nitrogen species (RNS) that are microbicidal and contribute to the control of infection^54–56^. Tissue-resident macrophages are directed toward an atypical M2 phenotype that is characterized by the expression of the chemokine receptor CXCL8 and cytokines such as TGF-β1 and IL-6. The arginine pathway in the M2 phenotype is directed by the upregulated expression of arginase to ornithine and urea which are precursors for proline and polyamine synthesis and are involved in tissue repair^56^.This antagonistic phenotype acts to “heal and fix” without killing the harboring *C. burnetii* promoting their survival and replication. During chronic infection, macrophages significantly reprogram toward the M2 phenotype. Although there is no conclusive evidence as to how this occurs, it is speculated that apoptosis of leukocytes in endocarditis results in their uptake by macrophages promoting the M2 phenotype that exhibits anti-inflammatory and immunosuppressive responses through the release of IL-10 and TGF-β^57,58^ facilitating the replication and dissemination of *C. burnetii*. M1 and M2 macrophages alleviate their specific responses, by directing T cells to differentiate into T_H_1 or T_H_2 lineage, respectively. The IL-4 and IL-10 cytokines produced by the M2 phenotype leads to the T_H_2 response, while IFN-γ and IL-12 produced by the M1 phenotype leads to the T_H_1 response. Subsequently, T cells continue to produce their respective cytokines, further driving macrophage effector functions in a positive feedback loop^55^. Macrophages derived from phase I immunized guinea pigs effectively degraded phase I *C. burnetii* even in the absence of immune serum^59^. Increased phagocytic activity without the involvement of antibodies indicates T cell-dependent activation. Macrophages, in addition to their role as phagocytes, present antigens in the form of peptide MHC-II complex for T cell activation^60^. The significance of this mechanism was underscored by the absence of protection in phase I-vaccinated mice lacking MHC-II molecules when subjected to *C. burnetii* infection^61^. Furthermore, the potent activation of macrophages commences with Toll-like receptor (TLR) recognition of pathogenic components, inducing transcription pathways that release mediators to prime adaptive immune responses. Studies on TLR2-deficient mice have demonstrated defective production of pro-inflammatory cytokines, such as TNF-α and IL-12 upon *C. burnetii* infection^62^. IL-12 promotes T cell differentiation to T_H_1 cells and exhibits synergistic effects with TNF-α for IFN-γ mediated killing of *C. burnetii*^63^. Hence, the protective mechanisms mediated by macrophages during *C. burnetii* infection and vaccination are a combined effort of the innate and cell-mediated immune responses.

### Neutrophils

As first responders to infection, they can recognize, ingest, and kill pathogens without adaptive responses. Phase I and II strains of *C. burnetii* infect neutrophils at a lower infection rate and infected neutrophils can further replicate and invade macrophages during apoptosis clearance of neutrophils^64^. An aerosolized infection model of SCID mice demonstrated delayed neutrophil influx at the infection site. This delay was explained by the reduction in pro-inflammatory cytokines attributed to *C. burnetii* immune evasion^64^. Neutrophil-depleted mice showed reduced body weight, increased splenomegaly, and increased bacterial burden upon infection with *C. burnetii*. This indicated their role in clinical disease reduction and bacterial clearance^65^. In contrast, phase I vaccinated and neutrophil-depleted mice displayed reduced body weight and splenomegaly with no difference in bacterial clearance compared to control mice. This suggests neutrophils play a role in vaccine-induced reduction of clinical signs, and not in bacterial clearance^65^.

### Dendritic cells

Dendritic cells (DC) or “alarm cells” are the immune sentinels that bridge the innate and adaptive immune responses. Their ability to acquire antigens and present them in the form of peptide MHC complexes to T cells orchestrates the cell-mediated responses to infections. In vitro studies with *C. burnetii* have shown that virulent phase I interferes with DC maturation. Human DCs infected with phase I expressed low levels of maturation markers such as MHC class II, CD80, CD86, CD83, and CD40 compared to the increased expression in phase II-infected DCs^66,67^. This study also demonstrated that phase II-induced DC maturation is TLR4-independent with increased production of IL-12 and TNF-α^67^. Contradicting this finding, Grovel et al. demonstrated the partial maturation of *C. burnetii* infected DCs with downregulated expression of TLR4, TLR3, STAT1, and interferon response genes^68^. This observation is further supported by a recent study in which, phase II infected DCs showed downregulated MHC expression with impaired maturation. However, the administration of IFN-γ reversed the inhibitory effects of phase II on DCs^69^. Many of these studies utilized monocyte-derived dendritic cells which have the disadvantage of poor IL-12p70 production, a heterodimer required for the activation and differentiation of T_H_1 cells^70^. Investigating the effect of different variants on DC subsets such as conventional DCs might shed more light on infection induced DC responses to *C. burnetii*. Several studies in murine models have shown the importance of DCs in vaccine-induced immunity. The chemokine receptor CCR7 directs DCs to lymph nodes for initiation of T cell responses^71^. The termination of phase I WCV induced cellular responses in *Ccr7*−*/−* mice, highlights the importance of DC trafficking to lymph nodes for initiation of cell-mediated responses^66^. In mice, the administration of phase II WCV-pulsed bone marrow-derived dendritic cells (BMDCs) led to a decrease in bacterial burden, demonstrating the protective role of antigen-activated BMDCs. This was linked to an increase in the proliferation of T_H_1 CD4^+^ helper T cells, skewed enhancement of T_H_17 cells, and a suppression of regulatory T cells^72^. T-bet is a transcription factor that is expressed in both lymphoid and myeloid lineages. T-bet is required by DCs for the production of IFN-γ, TNF-α, and activation of antigen-specific T cells^73,74^. T-bet knock out (KO) mice exhibited significant body weight loss and splenomegaly compared to phase I-vaccinated wild-type mice, indicating the essential role of DCs in initiating T cell-mediated responses^75^.

### Cell-mediated responses

**Fig. 1:**
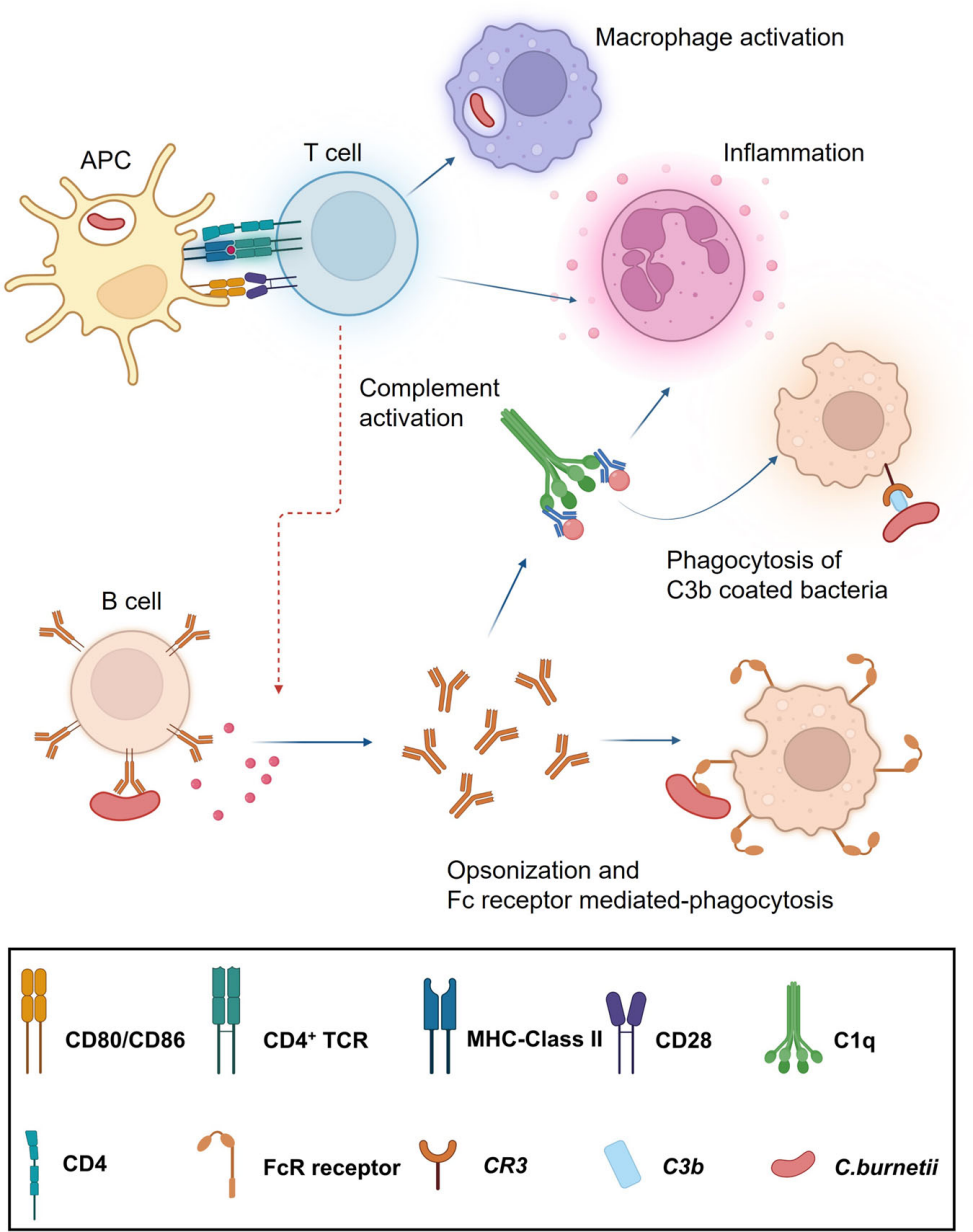
Cellular effector responses to *C. burnetii*. T cell responses are initiated upon peptide-MHC recognition by T cell receptor. Activated T cells differentiate to CD4^+^ helper T cells that secrete cytokines that activate B cells, macrophages, and inflammation. Activated B cells differentiate to plasma cell that produce antibodies. Antibody effector mechanisms include opsonization and Fc receptor mediated phagocytosis and complement activation.

The adaptive arm of immune defense marks the final defeat of the invading intracellular pathogens. Cell-mediated responses are primarily important for the clearance and control of *C. burnetii*, as they directly augment infected cells to boost their microbicidal activity (Fig. 1). Because of the intracellular nature of *C. burnetii* it is inaccessible to the effects of the humoral response^51^. Consequently, T cell-mediated killing of infected cells provides an important mechanism to prevent *C. burnetii* infection. The indispensable role of T cells in inducing cellular effector functions is demonstrated by the inability of severe combined immunodeficiency (SCID) mice to clear the infection with Nine Mile phase I (NMI) and phase II (NMII) strain^76^. Adoptive transfer of CD4^+^ and CD8^+^ T cells alone to infected SCID mice was sufficient to eliminate infection, conversely wild-type (WT) mice depleted of both T cells were susceptible to infection, whereas depletion of only one type of T cell, controlled infection^77^. SCID mice that received splenocytes and T cells from phase I immunized mice prevented splenomegaly and splenic bacterial burdens upon challenge, in addition to protection against the development of clinical disease and loss of body weight^78^. The initiation of the cellular cascade begins when *C. burnetii* is taken up by antigen-presenting cells at the site of infection. Dendritic cells in the mucosal airways are key mediators in shuttling *C. burnetii* from the site of infection (lungs) to the nearest lymphoid tissue to initiate T cell responses. In addition, *C. burnetii* can be disseminated to draining lymph nodes following infection^79^. Once in the mature and draining lymph nodes, activated and mature DCs present antigens in the form of an MHC class II peptide complex to naïve T cells. Upon infection with NMI, MHC II deficient mice exhibited translational loss in body weight, compared with WT mice^61^. Vaccination with inactivated NMI failed to reduce the bacterial load in MHC II KO and alum immunized mice^75^. Antigen-specific T cells proliferate and differentiate into effector CD4^+^ T cells. CD4^+^ T cells differentiate to the T_H_1 subset, which is largely determined by cytokines secreted by innate immune cells encountering an antigen. IFN-γ and IL-2 are characteristic cytokines produced by T_H_1 cells and are also the cytokines that drive their differentiation to T_H_1 subset. Individuals vaccinated with inactivated whole-cells exhibited lymphoproliferation and IFN-γ production suggesting a T_H_1 type response^78,80,81^. Upon encountering an infected macrophage, T_H_1 cells activate them to become more potent in their microbicidal functions. This classical activation is mediated by IFN-γ and co-stimulatory signals through the interaction of CD40L with CD40 on macrophages. IFN-γ activates the transcription factor STAT1, and CD40 signals activate nuclear factor-kappa beta (NF-κB). Together these induce lysosomal enzymes and the expression of phagocyte oxidase and inducible nitric oxide synthase (iNOS) enzymes that produce reactive oxygen (ROI) and nitrogen intermediates (RNI) respectively^25,26,82^. All these potent microbicidal agents help to kill *C. burnetii*, thus preventing their replication inside macrophages. The inability of iNOS^−/−^ and *p47phox*^*−/−*^ mice to control infection, upon challenge with *C. burnetii*, supports the role of RNI in host control of infection^83^. The vital role of IFN-γ in bacterial clearance has been demonstrated in IFN-γ^−/−^ mice models, and high-dose infection with NMI results in bacteremia and mortality during the early phase of infection^76^. In vitro studies with NMI infected THP-1 monocytes have revealed the ability of IFN-γ to reinstate the impairment of phagosome maturation^47^ and promote vacuolar alkalinization^37^. Infection of mouse L-292 cells with *C. burnetii* upregulates IFN- γ production, which augments iNOS expression^46^. Furthermore, IFN-γ activated macrophages from *iNOS*^*−/−*^ and *p47phox*^*−/−*^ mice were able to impede bacterial replication, indicating the potential of IFN-γ to activate iNOS- independent bacteriostatic mechanisms^83^. Although CD4^+^ T cell-dependent mechanisms have demonstrated their importance in *C. burnetii* clearance, Ledbetter et al. reported that CD4^+^-independent mechanisms play a more critical role in *C. burnetii* clearance in NMI-vaccinated mice^66^. The reduction in body weight, splenomegaly, and bacterial burden in MHC-I-deficient (*β2m* KO) mice compared to MHC II KO mice, indicates that protection by MHC I CD8^+^ T cells is crucial for *C. burnetii* clearance^61^. Furthermore, SCID mice reconstituted with CD4^+^ T cells alone exhibited increased bacterial burden and splenomegaly compared to mice reconstituted with CD8^+^ T cells^77^. This is likely due to the cytolytic activity of effector CD8^+^ T cells, which kill infected cells through the release of cytolytic compounds and induction of apoptosis in infected cells. While T cells are important for the primary clearance of *C. burnetii*, the role of B cells and their effector antibodies remains unclear. Antibody-mediated immunity includes, opsonization, complement-mediated killing and antibody‐dependent cellular cytotoxicity (ADCC)^84,85^. These effector functions are extracellular; hence, the role of antibodies for intracellular bacteria such as *C. burnetii* becomes crucial for the duration of time they are in the extracellular phase before inhabiting their target cells^85^. Vaccine-induced protection depends on B cell immunity, as indicated by increased splenomegaly and spleen bacterial loads in B cell-deficient mice compared to those in WT mice. This immunity is dependent on T cell-independent IgM, which inhibits *C. burnetii* infection in vivo^86^. Furthermore, this study demonstrated that antibody-mediated immunity is complement and FcR receptor-independent. This hypothesis is supported by a recent study in which FcR-KO, complement deficient, and WT mice were equally protected following passive immunization with mouse immune serum^87^. Previous findings where *C. burnetii* specific anti-serum added to macrophages failed to control their intracellular replication^88^ and the inability of macrophages to control antibody opsonized *C. burnetii*^87^ indicate a role for antibodies in initial clinical stages but not for clearance or elimination of infection. Schoenlaub et al. demonstrated that a population of B cells, B1a cells, might play a role in regulating the inflammatory response during *C. burnetii* infection. *C. burnetii* infected B1a cells induced high levels of IL-10 and TNF-α in vitro, whereas in vivo mice deficient in B1a cells (BTK^xid^) exhibited increased spleen inflammation, reduced IgM titers, and reduced levels of TNF-α^89^. However, these contradictory observations suggest that population of B cells might have a regulatory role in vitro, although this is insignificant in vivo.

## Q fever vaccine development: hits and misses

Since the discovery of the pathogen, many approaches have been taken to develop vaccines against Q fever, but no fully safe and effective vaccine has been developed to date. The only licensed vaccine currently available is Q-VAX, an inactivated whole cell vaccine derived from the Henzerling phase I strain. This vaccine is licensed for use only in Australia, and its worldwide administration is limited because of its reactogenic nature. Therefore, a pre-screening test including a skin test and serology is mandatory before administration. Owing to its restricted availability and safety, attempts to develop a safe vaccine are still an ongoing race with limited success. No vaccine has proven efficacy or afforded protection equivalent in humans to that of Q-VAX. To develop a successful vaccine, it is crucial to target, the concise immune effectors that are required to eliminate the pathogen and induce immunological memory to combat the pathogen in subsequent encounters without the establishment of disease. Most Q fever vaccines developed to date induce an immunological response but fail to confer protective immunity. These Q fever vaccine candidates can be categorized as whole cell vaccines or subunit vaccines and are summarized in Table 1.

**Table 1:** Summary of Q fever vaccines tested in humans or animal models.

Vaccine type	Human/animal model	Efficacy	Reactogenicity	References
Live attenuated vaccines				
M-44	Humans	Humoral response in 80% of individuals	Mild weakness, headache, temperature for 24 h	^91^
	Guinea pigs	Not reported	Myocarditis, hepatitis, necrosis, granuloma formation and splenitis	^92^
Whole cell Phase II	Mice	IgG response and similar protection to phase I WCV	Not reported	^93^
Killed whole cell vaccines				
Phase I WCV	Humans	Complement-fixing antibody titers equal to recovered patients	Redness at injection site, mild systemic reactions, malaise	^94^
		Protection for at least 5 years;Antibody and cell lymphoproliferative responses	Erythema, rarely Edema, abscess at injection site in few	^95,97^
	Guinea pigs	Complement-fixing antibodies	Local induration for a few days. Fever	^94^
* Δdot/icm*	Guinea pigs	Similar protection to WCV	Local erythema, local inflammation	^20^
	Ewes	Humoral response and protection demonstrated by reduced bacterial shedding and healthy lambs	No injection site reactions. Temperature rise post vaccination	^177^
	Goats	CD8^+^ IFN-γ response and IgG responseProtection was 33% more in adjuvanted WCV	Not reported	^178^
Phase II WCV	Ewes	Humoral response and protection demonstrated by reduced bacterial shedding and healthy lambs	No injection site reactions; temperature rise post vaccination	^177^
Soluble extracts				
CMR	Mice, guinea pigs, primates	Protection equivalent to Q-VAX	Local reactions	^104,105^
	Humans	IgG and lymphoproliferative responses	Local erythema and induration at injection site	^179^
TCA	Mice and guinea pigs	>90% protection with phase I and II antibodies	Local reactions	^102^
	Humans	Moderate humoral response	Local and systemic reactions	^102^
Soluble phase I	Mice, guinea pigs, non-human primates	Reduced bacterial burden in mice and guinea pigs	Reduced inflammation compared to WCV	^108^
Subunit vaccines				
TLR triagonists	Mice and guinea pigs	Protection depending on adjuvant formulation	Reactions with one	^117^
	Mice	Varying protection but not equivalent to Q-VAX	Not reported	^116^
O-Specific Polysaccharide/Tetanus Toxoid conjugate	Guinea pigs	Protection from fever and body weight loss. Reduced bacterial burden in organs	None	^18^
m1E41920-KLH (LPS mimetic)	Mice	Less protection than phase I LPS, but reduced splenic burden	Not reported	^118^

### Whole-cell vaccines

Whole-cell vaccines (WCV) can either be live attenuated or killed inactivated.

1. Live attenuated vaccines: one of the early vaccines developed for Q fever was the live attenuated vaccine M-44, which was used in Russia during the 1960s. This vaccine contains the Grita strain of *C. burnetii*, which was made avirulent by repeated passaging in embryonated yolk sacs^90^. Mass immunization of humans with live vaccine demonstrated mild reactogenicity following subcutaneous administration^91^. However, complications such as myocarditis, hepatitis, necrosis, and granuloma formation were identified 8 days post-infection in immunized guinea pigs, questioning its safety^92^. The use of avirulent viable NMII *C. burnetii* as a potential vaccine candidate was assessed in mouse models. This study demonstrated that intranasal immunization with NMII induced protection against NMI challenge, However, no information regarding reactogenicity was provided^93^.
2. Inactivated vaccines: to develop a refined Q fever vaccine, two inactivated vaccines derived from the Dyer or Henzerling strains were introduced in the 1980s. These vaccines are composed of formaldehyde-inactivated *C. burnetii* strains obtained from infected yolk tissues^94^. Evaluation of their efficacy in guinea pigs revealed a mortality rate of 40–80% in unvaccinated guinea pigs compared to 1–6% in vaccinated guinea pigs. When tested in humans, 3 out of 28 individuals developed mild systemic reactions, such as fever, anorexia, and malaise, with no serious reactions^94^. In mid-1981, clinical trials of inactivated Henzerling strains were conducted among abattoir workers and high-risk groups in South Australia to control Q fever^95^. At 18 months after vaccination, no Q fever cases were reported among 924 vaccinated subjects, whereas 34 cases were reported among 1349 unvaccinated subjects^96^. The trial revealed that vaccine conferred 100% protection in humans that lasted for at least 5 years. Assessment of cellular and humoral immunity revealed 80–82% seroconversion following vaccination, and lymphoproliferative responses in 85–95% of vaccinees^95,97^. The formalin-inactivated Henzerling strain vaccine; Q-VAX is currently approved for use only in Australia. The unsuccessful worldwide implementation is due to the occurrence of adverse effects in pre-sensitized individuals, primarily at the site of injection^98,99^. Data on adverse effects following immunization for Q-VAX were collected from clinical trials in abattoir workers^95,97^ and a mass vaccination program in the Netherlands following a Q fever outbreak^100^. These surveys revealed common reactions to vaccination such as erythema or induration at the injection site, with infrequent fever and transient headaches^95,96,100^. However, more severe reactions, such as delayed-type hypersensitivity reactions (DTH) were reported in individuals with prior exposure to *C. burnetii*^99,101,102^. For this reason, a pre-vaccination screening prior to vaccination is performed to determine pre-existing cellular or humoral immunity against *C. burnetii*. Recently, genetic engineering of whole-cell pathogens has been used, in which gene knockouts selectively remove the gene required for the pathogenicity of the organism rendering it avirulent and suitable for use as an attenuated vaccine. This also eliminates the risk of the organism reverting to its original virulent form. Based on this theory, a recent study assessed the immunogenicity and reactogenicity of a mutant version of the NMI strain in a guinea pig model. A 32 kb region encompassing 23 genes of the *dot/icm* system was deleted while retaining the phase I LPS. The resultant *Δdot/icm* mutant was avirulent in a guinea pig challenge model but demonstrated protective immunity following *C. burnetii* challenge with Δdot/icm sensitized guinea pigs. However, this vaccine displayed altered reactogenicity suggesting that the *C. burnetii* type IVB secretion system (T4BSS) is indispensable for vaccine-induced immunity but is a factor of post-vaccination hypersensitivity^20^. To further mitigate reactogenicity, whole-cell extracts of *C. burnetii* were explored for their potential as vaccines. In 1982 a chloroform-methanol extraction was used to extract the components of phase I Ohio strain. The extraction yielded a soluble phase consisting of lipids and a chloroform-methanol residue (CMR) composed of phase I LPS, proteins, and peptidoglycan^103^. When tested as a vaccine the CMR demonstrated good efficacy at a lower dose than Q-VAX in mice, and a dose four times higher than that of Q-VAX in guinea pigs, and a similar dose to Q-VAX in primates, but at the cost of local reactions^104–106^. A trichloroacetic acid (TCA) extract containing phase I antigenic components demonstrated more than 90% protection in *C. burnetii* challenged guinea pigs^102,107^. However, local reactions post-vaccination, such as transient erythema and local pain for several days at the site of injection have been reported with no severe abscesses^102^. Detergent extraction to obtain soluble antigens from the NMII avirulent strain yielded a soluble extract vaccine. When formulated with CpG adjuvant, this vaccine induced protective immunity in mice and guinea pigs against *C. burnetii* challenge with reduced erythema and induration at the site of injection compared to the whole-cell vaccine^108^.

Reactogenicity associated with WCVs is a result of innate and adaptive immune activation^109^. The recognition of conserved bacterial ligands called “pattern associated molecular patterns” (PAMPs) by pattern recognition receptors (PRRs) present on immune cells induces the production of inflammatory mediators that drive the adaptive response. During the initial exposure to *C. burnetii* this concomitant stimulation is favorable for pathogen clearance. However, during subsequent exposure in the form of vaccination or re-infection, the activation of adaptive responses together with innate recognition leads to an injurious cytokine-mediated inflammation called DTH reactions, which are mainly mediated by CD4^+^ T cells^110^. This reaction manifests as induration and swelling and occurs 24–48 h after antigen exposure. Clinical evaluation of guinea pig models following sensitization and immunization with C. *burnetii* showed a type IV hypersensitivity reaction^101,111^. With WCVs, there should be a fine balance between safety and immunogenicity, and the pathogen in the formulation should be virulent enough to induce immunological memory but less reactogenic to not elicit DTH reactions during subsequent exposure in the form of infection or vaccination.

### Subunit vaccines

Subunit vaccines are composed of immunodominant antigenic components that can be recombinantly produced or purified from microbes^112^. They offer the advantages of reduced reactogenicity and cost-effective production over traditional WCVs^113^. To augment immunogenicity, they are fused with a carrier or formulated with an appropriate adjuvant and administered in a multi-dose regimen^113–115^. A subunit vaccine composed of the outer membrane protein of *C. burnetii*; Com1 antigen formulated with TLR triagonist was evaluated for immunogenicity and reactogenicity in a mouse model. Although it induced a strong IgG2c skewed response it only conferred partial protection against *C. burnetii* compared to Q-VAX^116^. Fratzke et al. designed a subunit vaccine using six *C. burnetii* antigens formulated with multiple TLR agonists. Of the various vaccine formulations with different TLR agonists, only one vaccine containing TLR4, TLR7, and TLR9 agonists as triagonists demonstrated reduced reactogenicity and comparable bacterial burden to WCVs^117^. The findings from these studies emphasize the use of an appropriate adjuvant for subunit vaccine development to induce the desired immune response required for *C. burnetii* clearance. Phase I LPS (LPSI) of *C. burnetii* is considered a virulent determinant and most studies have explored LPS I as a vaccine candidate. The immune response induced by LPSI reduced *C. burnetii* loads in the spleen of intraperitoneally challenged mice but remained high in the spleens of mice after aerosol challenge^103^. An LPSI mimetic peptide m1E41920 screened using an LPSI monoclonal antibody (1E4) was conjugated to keyhole limpet hemocyanin (KLH) and tested in a mouse model. Although this combination induced transferable humoral responses, its protective efficacy was less than that of LPSI-immunized mice^118^. Recently a human T cell-directed epitope vaccine based on human leukocyte antigen (HLA) Class II T cell epitopes of *C. burnetii* was designed and evaluated in three animal models. Although the vaccine candidates induced a non-reactogenic response in a sensitized guinea pig model, the immune response elicited in mice was skewed toward a single epitope, which was deficient in conferring protection against *C. burnetii* challenge^81^. In addition, a tetanus toxoid carrier conjugated to a polysaccharide derived from phase I *C. burnetii* was recently developed as an alternative vaccine to Q-VAX. This vaccine demonstrated reduced febrile responses with reduced body weight loss in vaccinated guinea pigs post challenge with *C. burnetii*. Assessment of *C. burnetii* loads in the liver, spleen and kidney showed a significant reduction in vaccinated guinea pigs compared to unvaccinated controls^18^. One drawback of subunit vaccines is inadequate protective responses required for *C. burnetii* clearance. Identification of immunodominant antigens and selection of epitopes are crucial factors in designing subunit vaccines and require more knowledge beyond immunoinformatics.

## Moving forward: immunological targets for a better Q fever vaccine?

The current hurdle in Q fever vaccine development is the failure to generate long-lived immunological protection that prevents re-infection or disease severity. A vaccine that is equally efficacious, but less reactogenic than Q-VAX, remains the benchmark for developing an ideal Q fever vaccine. Approaches to implement this objective require an understanding of the precise immune mechanisms involved in the clearance of *C. burnetii* and the development of vaccines that elicit such responses at a certain threshold. Owing to its ability to build an intracellular niche *C. burnetii* requires T cell-driven adaptive responses for control and clearance. The induction of long-lived immunological memory requires the generation of memory T cells (both CD4^+^ and CD8^+^) and plasma B cells that secrete high affinity antibodies. To this end, we focused on vaccine strategies that warrant the induction of antigen-specific effector memory T or B cells, for better Q fever vaccine development.

### Programming T-cell responses

**Fig. 2:**
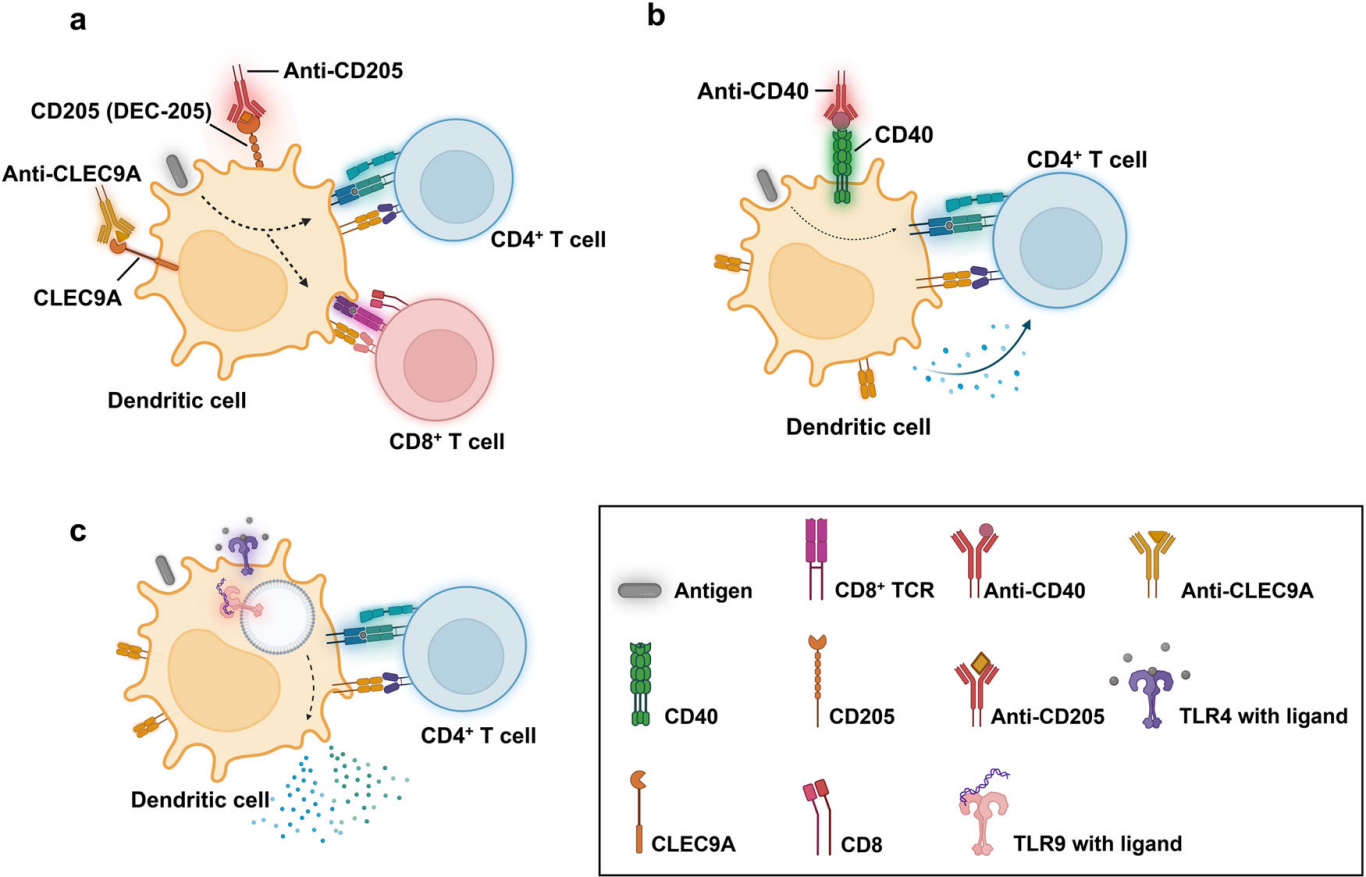
Programming T cell responses by targeted antigen delivery to dendritic cells (antigen-presenting cell). **a** Administration of anti-CD205 or CLEC9A with antigens, induces DC maturation and enhanced cross-presentation. **b** Anti-CD40 induces DC maturation, expression of costimulatory molecules and cytokines for T cell differentiation. **c** Co-delivery of TLR ligands with antigens: TLR9 present on the endosomal membrane detects unmethylated CpG DNA, TLR4 present on the cell membrane detects bacterial LPS. This innate recognition by pattern recognition receptors, in conjunction with antigen stimulation drives T cell activation.

The three phases of T cell response include activation, differentiation, and induction of memory. Immunological memory depends on the quantity and quality of memory T cells, which in turn depend on the magnitude of T cell expansion induced by a vaccine. The clonal expansion of T cells is modulated by T cell activation which is a collective effect of antigenic stimulation, co-stimulation, and polarizing cytokines released by innate mechanisms. These parameters can be influenced by the different forms of vaccination. During the activation phase, antigen recognition by TCR occurs through interaction with the peptide-MHC complex. Designing vaccines that effectively provide the unprocessed antigen to mature dendritic cells for exogenous or endogenous processing, and presentation to T cells via MHC molecules is an approach for targeted antigen delivery required for the activation phase (Fig. 2). Targeted delivery can be achieved by coupling antigens with immunomodulators that target the antigen-presenting cells. Approaches include linking antigens with antibodies against specific receptors present on APCs, for example, administration of anti-CD205 with antigens, induces CD205^+^(DEC-205) DCs a specific subset of cross-presenting DCs present in the spleen^119^. Additionally, antigen cross-presentation can be enhanced by co-delivery of anti-CLEC9A antibodies that target CLEC9A, a C-type lectin expressed exclusively by conventional dendritic cells^120,121^. Furthermore, the co-delivery of anti-CD40 promotes DC maturation through the interaction of CD40L on T cells. This interaction recruits the antigen-presenting cell to enhance expression of co-stimulatory molecules (B7-1, B7-2) and T cell differentiating cytokines such as IL-12, which further promotes potent activation of cognate T cell^122^. Adjuvants are an alternative form of immunomodulators that induce innate effectors to supplement adaptive responses. The use of PAMPs as adjuvants in vaccine formulations enables innate recognition of antigens, by PRRs^123^, resulting in the production of inflammatory cytokines that create an appropriate cytokine milieu for the activation and phenotypic differentiation of T cells. This approach has been explored in recent studies and has demonstrated protective immunity post-challenge with *C. burnetii*^116,117,124^. The incorporation of cytokines as adjuvants^125^ has yet to be explored as a vaccine approach for Q fever. A recent study revealed that IL-12 loaded nano-vaccine induced Hepatitis B virus-specific CD4^+^ and CD8^+^ T cell responses with long-term memory responses^126^. The incorporation of cytokines such as IL-12 or IFN-γ along with *C. burnetii* antigens as DNA vaccines could be explored as a promising Q fever vaccine. Collectively, these approaches trigger robust activation of T cells, which eventually become programmed for clonal expansion, followed by the contraction phase once the pathogen is cleared and eventually goes into induction of memory phase, the ultimate goal of a successful vaccine.

### Programming B-cell responses

**Fig. 3:**
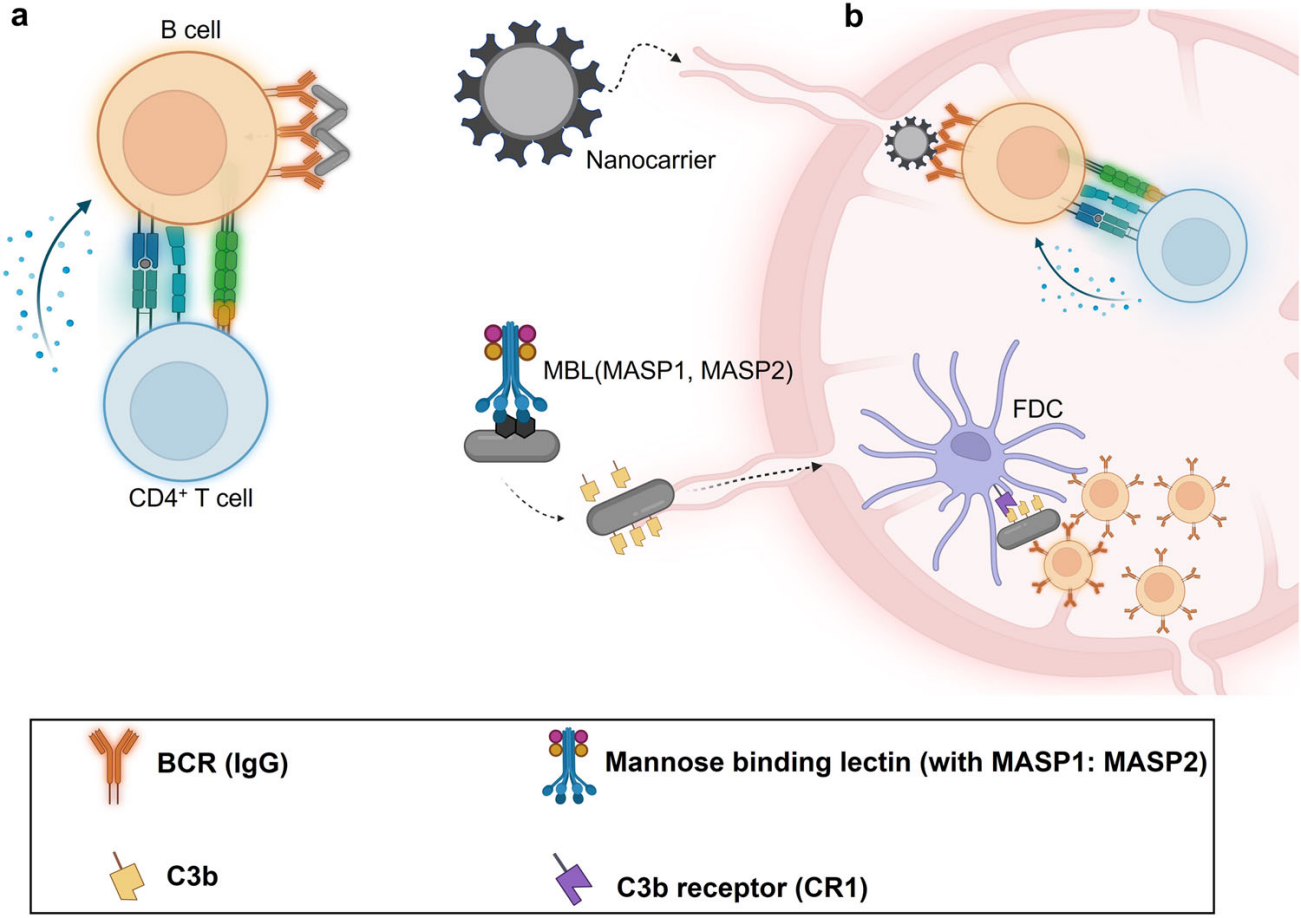
Programming B cell responses by driving germinal center (GC) reaction via potent B cell activation. **a** Multivalent antigens cause B cell receptor clustering that initiates signal transduction, which combined with costimulation from cognate T cells, amplifies B cell activation. **b** Nanocarriers help to unload antigen cargo in lymph nodes, they can be bioengineered for controlled release of antigens; glycosylated antigens activate the mannose-binding lectin complement pathway, that produces C3b, for antigen opsonization, which eventually binds to complement receptors in follicular dendritic cells. These cells display the antigens for recognition by B cells during the process of affinity maturation in the germinal centers.

Although intracellular *C. burnetii* remains inaccessible to antibodies, the role of humoral response in *C. burnetii* clearance and control becomes beneficial during the initial extracellular phase of *C. burnetii*. High affinity antibodies are produced in the germinal centers of lymphoid follicles during affinity maturation a process that selectively picks B cells with high affinity B-cell receptors (BCRs)^127^. B cell activation drives the germinal center (GC) reaction consisting of somatic hypermutation, affinity maturation, isotype switching, and generation of memory B cells^128^. Memory B cells upon subsequent antigen exposure, rapidly induce a secondary response, present antigens to memory T cells, and generate plasma cells within days feasibly before *C. burnetii* homes inside macrophages or monocytes^129,130^. Effective B cell stimulation requires antigen recognition and co-stimulation from cognate T cells. Activation of antigen-specific B cells is initiated by binding of antigen to BCR, which is internalized and presented as peptide-MHC complex to cognate T cells. In addition, it has been shown that APCs present antigens to B cells^131,132^. Targeted delivery of antigens to APCs can drive B cell activation similar to T cells, and this has been discussed in the previous section. Antigen valency is another factor that impacts B cell activation^133^. Multivalent antigens have demonstrated potent B cell signal transduction via BCR clustering compared to monovalent antigens^134,135^. Multiple antigens can be delivered by particulate carriers, which can also be fine-tuned to unload the antigen cargo in the lymphoid organs^136,137^. Targeting complement activation for antigen delivery to follicular dendritic cells (FDC) in the GC is an effective approach to drive GC reactions^138^. Glycosylated nanoparticles were shown to activate the complement system through mannose-binding lectin (MBL) pathway and subsequently deliver the antigen load to FDCs expressing complement receptors^139,140^. The FDCs present the antigens to B cells driving their affinity maturation during GC reaction. The various approaches are summarized in Fig. 3.

#### Eliminating vaccine-associated hypersensitivity

##### Threshold of activation

It has been described that CD4^+^ and CD8^+^ T cells have preferential requirements of co-stimulatory activation and distinct mechanisms in developing into effector T cells. CD8^+^ requires a lower threshold for activation as a consequence of universal MHC I. In contrast, CD4^+^ requires a high threshold of activation owing to the restricted expression of MHC class II and the need for co-stimulation by intrinsic ligands^141–143^. The DTH reactions induced in previously sensitized Q-VAX recipients are mediated by CD4^+^ T cells^111^. Induction of such responses during a secondary response in the form of vaccination depends on the supply and length of antigen exposure. Taking into consideration the high threshold of CD4^+^ activation, a WCV fulfills the antigen requirement for elicitation of high-frequency DTH CD4^+^ T cell responses. Switching the complete pathogen cargo in the form of the WCV to immunodominant antigens as subunit or particulate vaccines might mitigate the amplitude of aggressive CD4^+^ T cell responses while sustaining a sufficient threshold for CD4^+^ T activation^137,144,145^.The subcutaneous route of administration is known to retain antigens at the site of injection, which amplifies the recruitment of CD4^+^ T cells and cytokines to the site of injection. As *C. burnetii* transmission occurs by inhalation of SCV, induction of mucosal immunity by intranasal routes of administration paves an alternative approach to eliminating hypersensitivity reactions. This route could also prevent *C. burnetii* entry to cells through IgA-mediated effector functions^146,147^.

##### Vaccine components

Post-vaccination hypersensitivity reactions can result from individual vaccine components, including egg proteins, additives, and inactivators^110^. The use of formaldehyde to chemically inactivate *C. burnetii* may also contribute to Q-VAX-associated hypersensitivity. Although formaldehyde is removed from vaccines through purification processes, traces remain in the end-product^148,149^. It has been shown that formaldehyde-treated vaccines can cause reactive carbonyl groups to form on proteins, resulting in the development of novel immune-triggering epitopes or the unmasking of hidden immunogens that elevate non-specific immune responses^150,151^. Formaldehyde specific dermatitis have been reported following vaccination with Hepatitis B and influenza vaccines^152,153^. It is possible to eliminate formalin-associated hypersensitivity using alternate inactivation processes when considering a whole-cell vaccine for Q fever. Another probable factor contributing to hypersensitivity may stem from egg proteins present after purification of *C. burnetii* from chicken yolk tissues^154^. Studies on influenza vaccines have examined the safety of vaccines containing egg proteins, and indicates that vaccination of egg-allergic children with influenza vaccines does not pose a greater risk of allergic reactions^155^. However, not much data is available for egg-derived *C. burnetii*-based vaccines.

## Further considerations for effective Q fever vaccine development

In addition to identifying immunological targets and eliminating reactogenicity, it is imperative to consider other aspects of vaccine development for an improved Q fever vaccine, including (1) identification and selection of antigens, (2) vaccine delivery systems, (3) safety and scalability of antigen production, (4) screening for reactogenicity and (5) route of administration.

### Identification and selection of antigens

In recent years, the use of reverse vaccinology has enabled the identification of potential antigenic determinants in the *C. burnetii* genome. Many studies have focused on evaluating predicted antigens and epitopes as vaccine candidates in various animal models. Several immunoproteomic and protein microarrays have revealed sero-reactive *C. burnetii* antigens in murine and human sera^148–152^. Based on these findings, the identified antigens were screened for HLA class I and class II epitopes using bioinformatic analysis^153^. These epitopes were able to induce IFN-γ recall responses in HLA-DR3 transgenic mice and individuals exposed to a Q fever outbreak. The class II HLA epitopes identified in this study demonstrated robust T cell responses in patients with chronic Q fever as measured by direct ELISPOT, and these responses were comparable to those of convalescents^154^. Furthermore, another study evaluated the T cell epitopes of *C. burnetii* immunodominant antigens based on their MHC class II binding capacity and assessed their potential as T cell vaccines in mice^155^. Although protection was only established in mice immunized with a cocktail of epitopes, this study emphasizes the need for T cell epitopes to be present in vaccine candidates for the induction of protective immunity. A viral vector vaccine expressing concatemers of selected HLA class II epitopes demonstrated a single epitope-specific response in mice, whereas a broad range of epitope-specific responses was induced in cynomolgus macaques^81^. These findings emphasize the need to select antigens that elicit immune responses in a wide range of species. Besides protein antigens, *C. burnetii* phase I LPS has also been evaluated as a potential Q fever vaccine antigen^118^. Based on previous observations that LPS plays a major role in WCV-induced protection, LPS based vaccine candidates were recently developed and mediated significant reduction in clinical signs and bacterial burden implicating the involvement of phase I LPS in *C. burnetii* protective immune responses^18,156^. Xiong et al. reported novel MHC Class I epitopes from *C. burnetii* Type IV secretion system (T4SS) none of which were identified as immunodominant antigens through antibody-based methods^157^. These peptides were able to induce robust CD8^+^ T-cell recall responses in infected mice and conferred measurable protection when tested as potential antigen candidates via the *Listeria monocytogenes* vaccine vector. Alternatively, a recent study reported that the Type IVB secretion system (T4BSS) of *C. burnetii* is dispensable for vaccine -induced immunity but may contribute to vaccine-induced hypersensitivity^20^. Despite significant advances, the fact that similar systems of *C. burnetii* induce different responses in hosts reveals gaps in our understanding of *C. burnetti* antigens.

### Vaccine delivery systems

Delivery systems enable the targeted and controlled release of antigens to the site of the immune response. These systems can also be effectively tailored to induce the desired immune response by altering their physical characteristics such as size, geometry, and surface charge^158–160^. Delivery systems currently in use include biopolymers, liposomes, immune-stimulating complexes (ISCOMs), virus like particles (VLPs), and bacterial and viral vectors. A few studies have investigated the use of viral and bacterial vectors to incorporate *C. burnetii* antigens and have demonstrated the potential of these delivery systems to elicit cell-mediated responses^81,157^. Biopolymer-based vaccines have been evaluated as vaccine platforms for intracellular pathogens and have demonstrated the ability to elicit antigen-specific cellular responses that confer protective immunity against infection^161^. These studies are promising and encourage the use of delivery systems as an improved Q fever vaccine approach to eliminate reactogenicity and to boost desirable immune responses.

### Safety and scalability of antigen production

The laborious production of conventional WCVs carries the risk of handling *C. burnetii* which poses a threat to laboratory personnel and limits the scalability of production. Modern advancements have surmounted this barrier with alternative vaccine approaches, including nucleic acid-based vaccines, subunit vaccines, and gene knockout WCVs. *C. burnetii* protein production using recombinant technology has significantly reduced safety concerns. Protein expression systems, such as e.g., using safe *Escherichia. coli* as production host, have enabled scalable production of biopolymer- and protein-based vaccines^136,137,162^. These strategies provide alternative paths for the safe, cost-effective, and scalable production of Q fever vaccines.

### Screening for reactogenicity

The current approach for reactogenicity screening of experimental vaccines involves a sensitized animal model. Guinea pigs appear to be the primary model because of their physiological resemblance to human disease, while mice and non-human primates serve as complementary models^163^. The use of non-mammalian models, such as the *Galleria mellonella* insect model, has also been explored and overcomes the ethical and high cost limitations of mammalian models^164^. The complexity of the immune response coupled with a lack of understanding of the correlates of infection and vaccine-induced protection makes it difficult to define an ideal model for reactogenicity screening. Telemetric measurements to monitor systemic side effects of vaccination have been reported to be more useful than conventional clinical scoring methods^165^. Identification of potential biomarkers of vaccine induced responses can serve as predictors of systemic reactogenicity^166,167^. Immune markers such as C-reactive protein (CRP), IL-6, IFN-γ, IP-10, and MCP-2 were associated with systemic symptoms in individuals vaccinated with AS01_B_—Hepatitis B surface antigen^168^. Alternative approaches include metabolomics and transcriptomics, which can be used to predict markers of vaccine reactogenicity and accelerate the clinical testing of Q fever candidates^169^.

### Route of administration

*C. burnetii* whole-cell vaccines have traditionally been administered subcutaneously, and this route may be responsible for reactogenicity, even though it triggered protective immune responses. It would be worthwhile to explore other routes that might enhance or retain immunogenicity while reducing reactogenicity. As with subcutaneous routes, intramuscular routes induce similar responses, but with fewer local reactions^109^ The intramuscular route proved to be less adverse for whole-cell formulations with alum than the subcutaneous route. The CDC recommends the intramuscular route for inactivated vaccines formulated with adjuvants because it causes less inflammation and granulomas^170,171^. However, no studies have reported the intramuscular administration of *C. burnetii* WCVs with adjuvants; hence, the reactogenicity associated with this route remains unclear. Infectious diseases that spread through airborne transmission are well-suited for mucosal vaccines^172,173^. The induction of mucosal immunity leads to secretory IgA responses that neutralize pathogens at the point of entry^173,174^. This route has proven effective in subunit CMR vaccines, with no data on reactogenicity^175^. Depending on the vaccine platform, it is important to choose an ideal route that induces the maximum response without causing local or systemic reactions.

The primary goal is to develop a universal preventative Q fever vaccine. The ideal vaccine to combat this biological warfare agent is one that prevents infection and ultimately disease development. Therefore, a pre-infection vaccine that prevents acute infection and progression to a chronic state should be considered. From an immunological perspective, a pre-infection Q fever vaccine evokes a pre-existing immune response that intervenes in the pathogenesis of *C. burnetii* that occurs upon exposure. The chronic state of Q fever occurs due to the inability of the immune system to clear *C. burnetii*^76,77^. This is probably due to the delayed onset of the T cell response, which is initiated much later owing to the immune evasion mechanisms of *C. burnetii*^5,176^. The presence of pre-existing *C. burnetii*-specific CD4^+^ T cells, in combination with pre-existing antibodies at the time of exposure is a valid strategy to counteract evasion mechanisms. Additionally, an effective vaccine should evoke an efficacious response that confers protection upon subsequent exposure. To this end, future Q fever vaccine development efforts should consider a prevention of infection vaccine that induces long-lasting and memory immune responses in order to reduce the rate of disease transmission and thus control of disease among populations.

## Concluding remarks

Although extensive studies aiming at the development of non-reactogenic Q fever vaccines have been conducted, such a vaccine remains elusive. The current knowledge of immunology underpinning protective immunity against *C. burnetii* indicates that both humoral and cell-mediated immune responses are required for Q fever prevention. However, there are still certain knowledge gaps that must be addressed with regard to the development of a Q fever vaccine. An in-depth analysis of Q fever prevalence across different populations and regions is crucial to assess heterologous protection against different strains. There is still limited understanding of the pathogenesis and immune mechanisms induced by *C. burnetii*, which makes it difficult to elucidate correlates of protection required for vaccine development. Identifying optimal antigenic determinants as vaccine targets is central to vaccine discovery, and information in this area remains obscure despite advancements in bioinformatics. Furthermore, to date, no studies have reported the long-term durability and longevity of responses following vaccination. A comprehensive approach to these research questions should provide useful information for precise engineering of future Q fever vaccines.
